# Neurocognitive abilities in the general population and composite genetic risk scores for attention-deficit hyperactivity disorder

**DOI:** 10.1111/jcpp.12336

**Published:** 2014-10-03

**Authors:** Joanna Martin, Marian L Hamshere, Evangelia Stergiakouli, Michael C O'Donovan, Anita Thapar

**Affiliations:** 1 MRC Centre for Neuropsychiatric Genetics and Genomics, Institute of Psychological Medicine and Clinical Neurosciences, Cardiff UniversityCardiff, UK; 2 MRC Integrative Epidemiology Unit, University of BristolBristol, UK

**Keywords:** ALSPAC, ADHD, genetics, cognition

## Abstract

**Background:**

The genetic architecture of ADHD is complex, with rare and common variants involved. Common genetic variants (as indexed by a composite risk score) associated with clinical ADHD significantly predict ADHD and autistic-like behavioural traits in children from the general population, suggesting that ADHD lies at the extreme of normal trait variation. ADHD and other neurodevelopmental disorders share neurocognitive difficulties in several domains (e.g. impaired cognitive ability and executive functions). We hypothesised that ADHD composite genetic risk scores derived from clinical ADHD cases would also contribute to variation in neurocognitive abilities in the general population.

**Methods:**

Children (*N* = 6,832) from a UK population cohort, the Avon Longitudinal Study of Parents and Children (ALSPAC), underwent neurocognitive testing. Parent-reported measures of their children's ADHD and autistic-like traits were used to construct a behavioural latent variable of ‘neurodevelopmental traits’. Composite genetic risk scores for ADHD were calculated for ALSPAC children based on findings from an independent ADHD case–control genome-wide association study. Structural equation modelling was used to assess associations between ADHD composite genetic risk scores and IQ, working memory, inhibitory control and facial emotion recognition, as well as the latent ‘neurodevelopmental trait’ measure.

**Results:**

The results confirmed that neurocognitive and neurodevelopmental traits are correlated in children in the general population. Composite genetic risk scores for ADHD were independently associated with lower IQ (*β *= −.05, *p* < .001) and working memory performance (*β *= −.034, *p* = .013), even after accounting for the relationship with latent neurodevelopmental behavioural trait scores. No associations were found between composite genetic risk scores and inhibitory control or emotion recognition (*p* > .05).

**Conclusions:**

These findings suggest that common genetic variants relevant to clinically diagnosed ADHD have pleiotropic effects on neurocognitive traits as well as behavioural dimensions in the general population. This further suggests that the well-recognised association between cognition and neurodevelopmental behavioural traits is underpinned at a biological level.

## Introduction

Attention-deficit hyperactivity disorder (ADHD) is a highly heritable neurodevelopmental disorder (Faraone et al., 2005). It is clear that the genetic architecture of ADHD is complex, with common and rare variants involved (Neale et al., 2010; Stergiakouli et al., 2012; Williams et al., 2010; Yang et al., 2013). ADHD is strongly associated with autism spectrum disorder (ASD) and other neurodevelopmental disorders (Thapar, Cooper, Eyre, & Langley, 2013). Twin studies also demonstrate significant co-heritability across neurodevelopmental disorders and traits, including ADHD and ASD (Lichtenstein, Carlström, Råstam, Gillberg, & Anckarsäter, 2010; Ronald, Simonoff, Kuntsi, Asherson, & Plomin, 2008). These findings suggest that genetic risk variants for ADHD are likely to contribute to multiple neurodevelopmental behavioural traits and disorders.

Indeed, there is an increased burden of rare copy number variants (CNVs) in ADHD, which overlap more than expected by chance with regions implicated in other neurodevelopmental conditions, such as ASD or schizophrenia (Lionel et al., 2011; Martin et al., 2014a; Williams et al., 2010, 2012). However, such CNVs are rare and so it is likely that other types of genetic variants also contribute to this overlap.

There is evidence that commonly occurring genetic variants play a role in risk for ADHD. First, when considered in aggregate (i.e. as a composite risk score), common risk alleles defined from a clinical ADHD case-control genome-wide association study (GWAS) are higher in an independent set of ADHD cases than controls (Hamshere et al., 2013a). Second, common additive variants collectively show moderate heritability for ADHD when estimated using genome-wide complex trait analysis (GCTA) software (Lee et al., 2013; Yang et al., 2013).

The evidence is mixed so far in terms of possible overlap of common genetic risk variants across ADHD and other disorders. One study found that common risk alleles relevant to schizophrenia are enriched in ADHD cases when compared with controls (Hamshere et al., 2013b). However, two recent cross-disorder studies from the Psychiatric Genomics Consortium, found no overlap of common variants across clinical samples of ADHD and ASD or schizophrenia (Lee et al., 2013; Smoller et al., 2013). Nevertheless, these analyses are inconclusive as the ADHD and ASD samples were substantially smaller than those available for the adult disorders, which did show significant overlap of risk alleles. Furthermore, in a general population cohort, composite genetic risk for ADHD, using scores derived from a clinical discovery sample, predict ADHD and social-communication/autistic-like behavioural traits (Martin, Hamshere, Stergiakouli, O'Donovan, & Thapar, 2014b). On the whole, these results suggest that common genetic variants may show some pleiotropic effects on different neurodevelopmental behavioural traits, both in clinical cases and in the general population.

### Neurocognitive problems

Children affected by ADHD often have multiple, prominent neurocognitive difficulties. These affect a range of domains, notably involving global cognitive ability (Frazier, Demaree, & Youngstrom, 2004) and executive functioning, such as response inhibition and working memory (Willcutt, Sonuga-Barke, Nigg, & Sergeant, 2008), as well as aspects of social cognition, such as facial emotion recognition (Collin, Bindra, Raju, Gillberg, & Minnis, 2013; Uekermann et al., 2010). Like ADHD, these neurocognitive domains are heritable (Ando, Ono, & Wright, 2001; Deary, Johnson, & Houlihan, 2009; Rommelse, Geurts, Franke, Buitelaar, & Hartman, 2011). There is also evidence suggesting that difficulties in these domains share familial and genetic risks with ADHD (Bidwell, Willcutt, DeFries, & Pennington, 2007; Kuntsi et al., 2004; Schachar et al., 2005). Moreover, difficulties in these domains are not unique to ADHD, but rather these deficits are also strongly associated with other neurodevelopmental disorders, including ASD and schizophrenia (Fett et al., 2011; Matson & Shoemaker, 2009; Uljarevic & Hamilton, 2012; Willcutt et al., 2008). It is not yet known whether common genetic variants relevant to clinical ADHD also contribute to neurocognitive abilities, typically assessed through task-based performance, in the general population.

The aim of the current study was to examine whether a common genetic composite risk score, based on case-control GWAS findings for clinical ADHD, influences general cognitive ability, executive functioning and social cognition in children from the general population. It was hypothesised that composite genetic risk scores would predict lower neurocognitive abilities in these domains. A secondary aim was to test whether observed associations were independent of associations between neurocognitive abilities and neurodevelopmental behavioural traits, specifically, parent-reported ADHD and social-communication behavioural traits in this sample (Martin et al., 2014b).

## Methods

### Target population sample – ALSPAC

The Avon Longitudinal Study of Parents and Children (ALSPAC) is a large, well-characterised longitudinal dataset (Boyd et al., 2013; Fraser et al., 2013). ALSPAC originally recruited *N* = 14,541 pregnant women resident in Avon, England, with expected delivery dates of April 1, 1991, to December 31, 1992. Of these pregnancies, *N *= 13,988 children were alive at age 1 year. An additional eligible 713 children were enrolled after age 7, resulting in a total sample of *N* = 14,701 of children. The study website (http://www.bris.ac.uk/alspac/researchers/data-access/data-dictionary/) contains details of all available data. Ethical approval for the study was obtained from the ALSPAC Ethics and Law Committee and the Local Research Ethics Committees. Full data (phenotypic and genotypic) were available for up to *N* = 6,832 children, depending on the outcome variables.

### Neurocognitive task measures

ALSPAC families were invited to attend a ‘Focus’ clinic when the children were aged approximately 8.5 years old, where they underwent neuropsychological testing. A short form of the WISC-III assessment was employed to obtain an estimate of full scale IQ (Wechsler, 1992). In addition to this, the WISC-III Digit Span task was administered to obtain a measure of verbal working memory. Cognitive inhibitory control was assessed using the Opposite Worlds task from the Tests of Everyday Attention for Children battery (Robertson, Ward, Ridgeway, & Nimmo-Smith, 1996). The mean time taken to complete the control condition (Same Worlds trials) was subtracted from the mean time for the experimental condition (Opposite Worlds trials) and the resulting score was transformed (1/square root of score). Facial emotion recognition was assessed using a computerised version of the faces subtest of the Diagnostic Analysis of Nonverbal Accuracy (DANVA) (Nowicki & Duke, 1994). The task comprised four emotion types (happy, sad, angry or fearful) shown at high (easy condition) and low (hard condition) intensities. The total number of errors made on the 12 low emotional intensity faces (normally distributed) was used for analysis.

In addition to these four measures (IQ, working memory, cognitive inhibitory control and facial emotion recognition), the Counting Span Task, another measure of working memory, was administered at age ∽10.5 years. The measure used was a computer-generated normally distributed ‘global score’, corresponding to the number of correct trials.

### Parent-reported measures of ADHD and social-communication

Measures of ADHD and social-communication assessed at a similar time to the neurocognitive measures were included in analyses as these were previously found to be associated with ADHD composite genetic risk scores (Martin et al., 2014b). Total ADHD inattentive and hyperactive-impulsive traits were calculated by summing the relevant items from the parent Development and Well-Being Assessment (DAWBA) administered at approximately 7.5 years old (Goodman, Ford, Richards, Gatward, & Meltzer, 2008). Social-communication traits were assessed using the Social and Communication Disorders Checklist (SCDC) at the same age (Skuse, Mandy, & Scourfield, 2005), as well as the pragmatic language scales of the Children's Communication Checklist (CCC) at approximately 9.5 years (Bishop, 1998). CCC pragmatic language scores were transformed (ln × + 1) and reversed so that higher scores meant more difficulties.

### Genetic data

A total of 9,912 ALSPAC children were genotyped. After quality control (QC), genome-wide data for 500,527 single nucleotide polymorphisms (SNPs) were available for *N* = 8,229 of the children, of whom *N* = 4,213 (51.2%) were male. Details of QC procedures have been reported previously (Martin et al., 2014b).

The results of a published GWAS of British and Irish children with a diagnosis of ADHD (*N* = 727) and population controls (*N* = 5,081) were utilised as the primary discovery sample. The QC procedures, ascertainment of these samples and GWAS results have been described in detail previously (Stergiakouli et al., 2012). This GWAS was based on 502,702 SNPs after QC. Composite risk scores were calculated for each ALSPAC child using PLINK (Purcell et al., 2007), based on the results of the above GWAS discovery sample, as described previously (Martin et al., 2014b). In brief, SNPs in approximate linkage equilibrium in the ALSPAC data were identified using PLINK, with SNPs exceeding a threshold of *R*^2^ ≥ .2 excluded. As in our previous work, the primary tests of our hypotheses were based on risk alleles enriched (at *p* < .5) in ADHD cases in the discovery GWAS. This list of risk alleles was used to calculate a composite risk score for each individual in ALSPAC, corresponding to the mean number of score alleles (weighted by odds ratio) across the set of SNPs, using the PLINK command (–score). Composite risk scores were also calculated at a variety of other *p*-value thresholds to test the sensitivity of observed results. The composite genetic scores were standardised using *z*-score transformations.

### Data analysis

Pearson correlations were used to examine associations between performance on the neurocognitive measures and parent-reported ADHD and social-communication traits. Females and males were compared in terms of neurocognitive phenotypes, using Student's *t*-test. The neurocognitive measures were standardised using *z*-score transformations. Analyses were performed using Stata version 13.0 (StataCorp., 2013).

The main analysis was conducted in several steps. First, multivariate linear regression analyses were used to test for associations between composite genetic risk scores and each of the neurocognitive measures, after controlling for the effect of gender.

Next, given previous work showing that ADHD composite genetic risk scores predict both parent-reported ADHD and social-communication traits (Martin et al., 2014b), confirmatory factor analysis was used to derive a ‘neurodevelopmental difficulties’ latent variable. This variable was based on parent-reported ADHD inattentive and hyperactive-impulsive traits, as well as CCC pragmatic language and SCDC social cognition scores. Goodness of fit of the latent variable model was assessed using the root mean square error of approximation (RMSEA), comparative fit index (CFI) and Tucker-Lewis fit index (TLI) statistics. Good model fit was indicated by an RMSEA fit statistic ≤ .06 and CFI and TLI statistics > .95 (Hu & Bentler, 1999). Multiple indices were used as this provides a more comprehensive evaluation of model fit.

As a final step, structural equation modelling (SEM) was used to test a model of ADHD composite genetic risk score effects on neurocognitive measures and the latent variable of neurodevelopmental difficulties. SEM has the potential to simultaneously estimate the relationships of multiple manifest/observed and latent predictor and outcome variables. For the purpose of this study, the SEM models combine regression, path and factor analyses. Given that neurodevelopmental and neurocognitive measures are related, modelling the association of composite genetic risk scores with both measures simultaneously can be used to determine the unique effect of composite genetic scores on each of these measures. On figures, manifest variables are represented by squares, latent variables are represented by circles, single direction arrows indicate regression paths, double direction arrows indicate correlations and numbers indicate regression/correlation coefficients.

Model goodness of fit was assessed as above. The model estimator used was ‘MLR’, which is robust to non-normality and provides full information maximum likelihood estimation with robust standard errors, using all available data for each model. SEM was performed using Mplus version 7 (Muthén & Muthén, 1998). All *p*-values presented are two-tailed. Given the non-independence of the four neurocognitive measures and the structured analytic approach, all results are interpreted using a significance threshold of *p* < .05.

### Secondary analyses

Analyses were re-run using 10 EIGENSTRAT principal components as covariates to account for possible population ancestry effects. Analyses were also re-run using composite risk scores calculated in ALSPAC using alternative p-value selection thresholds.

### Replication analyses

Where significant associations were observed, comparable analyses were run to determine whether the same associations in the ALSPAC sample could be replicated using composite risk scores derived from an independent ADHD discovery sample (using the same analytical method). This second sample consisted of the published international Psychiatric Genomics Consortium (PGC) meta-analysis of ADHD case-control GWAS (Neale et al., 2010) based on four individual studies. It consisted of 2,064 trios, 896 cases and 2,455 control individuals and 1,206,461 SNPs after QC.

## Results

### Phenotypic relationships

Table1 shows Pearson correlation coefficients for associations between each neurocognitive measure and ADHD and social-communication trait scores. All correlations are significant (*p* < .001), although coefficients are low to modest, with the highest correlation being between IQ and the Digit Span subtest (*r* = .39).

**Table 1:** Correlations between neurocognitive and neurodevelopmental measures

	IQ	Working memory	Inhibitory control	Emotion recognition
Working memory	.39			
Inhibitory control	.21	.11		
Emotion recognition	−.16	−.11	−.09	
ADHD hyperactive-impulsive	−.16	−.13	−.07	.09
ADHD inattentive	−.21	−.19	−.12	.09
Social cognition	−.14	−.11	−.05	.10
Pragmatic language	−.25	−.18	−.06	.08

All significant at *p* < .001.

Males had lower working memory scores (age 8.5 years: *t* = −6.79, *p* < .001; age 10.5 years: *t* = −2.30, *p* = .022) and made more emotion recognition errors (*t* = 4.46, *p* < .001) than females. They did not differ from females in terms of inhibitory control (*t* = −1.78, *p* = .075) or IQ (*t* = .68, *p* = .50).

### Neurocognition and composite genetic scores

After adjusting for gender, ADHD composite genetic risk scores, derived from the primary discovery sample (Stergiakouli et al., 2012), predicted lower IQ (*β *= −.05, *p* < .001, *R*^2^ = .0027) and lower working memory abilities (*β *= −.034, *p* = .013, *R*^2^ = .0011). There were no associations with inhibitory control (*β *= .006, *p* = .66) or emotion recognition (*β *= −.004, *p* = .76).

**Figure 1:**
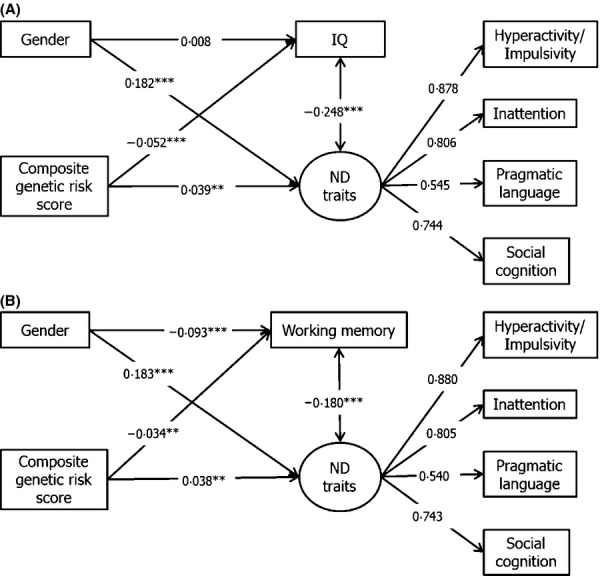
Associations between composite genetic scores with IQ and working memory. **p < .01, ***p < .001; ND: Neurodevelopmental. (A) Association of composite genetic scores with IQ (*N* = 6,832); RMSEA = .053, CFI = .974, TLI = .952. (B) Association of composite genetic scores with working memory (*N* = 6,827); RMSEA = .045, CFI = .981, TLI = .966

The confirmatory factor analytic/latent variable model of inattentive, hyperactive-impulsive, social-cognitive and pragmatic language traits showed moderately good fit (RMSEA = .053, CFI = .994, TLI = .982; see Figure S1). SEM testing was used to simultaneously model the associations of ADHD composite genetic risk scores with the latent neurodevelopmental trait variable and the neurocognitive measures, as well as the correlation between these measures. The SEM results were consistent with the linear regression analyses, with higher ADHD composite genetic risk scores predicting lower IQ (*β *= −.052, *p* < .001, *R*^2^ = .003) and working memory (*β *= −.034, *p* = .008, *R*^2^ = .001) and no associations with inhibitory control and facial emotion recognition (*p* > .05). Model diagrams are displayed in Figure1 and Figure S2. Model fit was satisfactory for all models (see figure captions).

**Figure 2:**
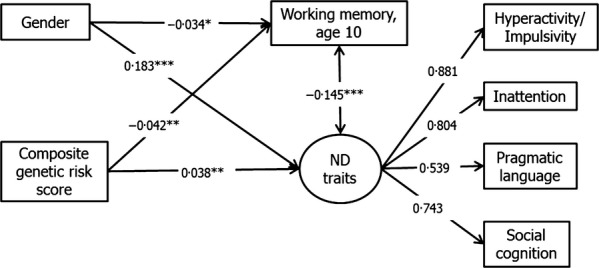
Association between composite genetic scores with working memory at age 10 years. *p < .05, **p < .01, ***p < .001; ND: Neurodevelopmental. *N* = 6,847; RMSEA = .045, CFI = .981, TLI = .966

ADHD composite risk scores also predicted lower working memory abilities (*β *= −.042, *p* = .002, *R*^2^ = .0018), as assessed using the global score of the Counting Span Task at the approximate age of 10.5 years. SEM with the neurodevelopmental difficulties latent variable showed a consistent result (*β *= −.042, *p* = .002, *R*^2^ = .002); see Figure2.

### Secondary analyses

SEM including both IQ and working memory (at age 8.5 years) as correlated outcomes showed that these outcomes are independently predicted by ADHD composite genetic risk scores (see Figure S3).

To test the sensitivity of the results with regards to inclusion of the latent neurodevelopmental variable, analyses were re-run with ADHD inattentive and hyperactive-impulsive traits as manifest outcome variables and omitting the measures of social-communication. The pattern of results was the same as before (see Figure S4).

To look at the effect of population stratification, analyses were co-varied for 10 EIGENSTRAT covariates. This did not affect the results (see Table S1). The pattern of results was consistent across different selection thresholds for generating the composite genetic risk scores (see Figure S5).

Gender was entered as a covariate for all models. When analyses were instead stratified by gender, the association between composite genetic risk scores and IQ was seen separately for both females (*β *= −.060, *p* = .0011, *R*^2^ = .0038) and males (*β *= −.045, *p* = .023, *R*^2^ = .0019), whereas the association with working memory at age 8 was only seen in females (*β *= −.041, *p* = .031, *R*^2^ = .0017) and not males (*β *= −.027, *p* = .16, *R*^2^ = .0007).

### Replication analyses

**Figure 3:**
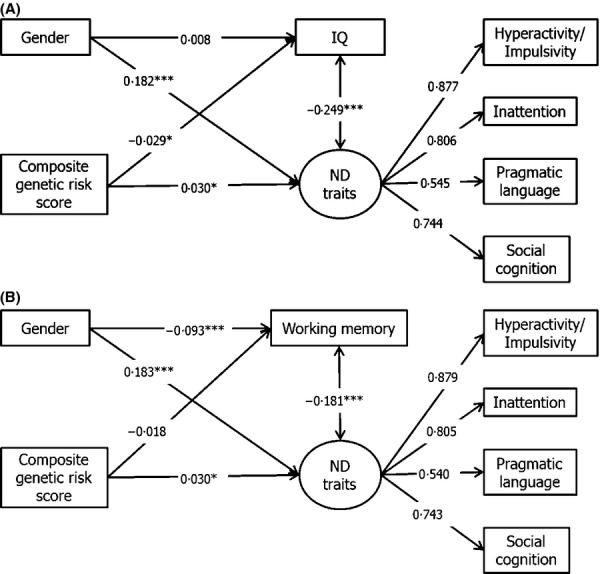
Associations between composite genetic risk scores (based on replication discovery sample) with IQ and working memory. **p < .01, ***p < .001; ND: Neurodevelopmental. (A) Association of composite genetic scores with IQ (*N* = 6,832); RMSEA = .053, CFI = .974, TLI = .953. (B) Association of composite genetic scores with working memory (*N* = 6,827); RMSEA = .045, CFI = .982, TLI = .967

Composite risk scores derived from the published replication discovery data (Neale et al., 2010) showed an association with lower IQ (*β *= −.030, *p* = .028, *R*^2^ = .0009), but no association with working memory at either time point (age 8: *β *= −.018, *p* = .18; age 10: *β *= −.015, *p* = .27). SEM testing showed the same pattern of results (see Figure3 and Figure S6).

## Discussion

This study finds that common genetic risk variants that predict risk of clinical ADHD diagnosis and that were previously found to predict parent-reported ADHD and social-communication behavioural traits in the general population (Martin et al., 2014b) are also associated with lower IQ and working memory in children from the general population. ADHD composite genetic risk scores were not found to be associated with inhibitory control or facial emotion recognition measures. The associations of ADHD composite genetic risk scores with IQ and working memory persisted even after taking into account associations with neurodevelopmental (ADHD and social-communication) behavioural traits, using structural equation modelling, and were robust to sensitivity testing. These results suggest that common genetic risk variants relevant to a clinical diagnosis of ADHD may have effects on multiple neurocognitive abilities, as well as behavioural traits in the general population.

The association between ADHD composite genetic risk scores and working memory was also observed with a different measure of working memory, assessed at a later time point. Composite genetic risk scores based on changing the allele selection thresholds were also consistently associated with both IQ and working memory problems at both time points. The observed association between ADHD composite genetic risk scores and IQ was further robust to replication using a second ADHD discovery dataset (Neale et al., 2010). However, no association was observed with either measure of working memory using composite genetic risk scores derived from the replication discovery data.

This inconsistency of results using the primary and replication discovery samples could theoretically be attributable to potential differences in clinical or cognitive profiles between the two datasets (Neale et al., 2010; Stergiakouli et al., 2012). Alternatively, subtle differences in ancestry may have contributed to this discrepancy; the primary discovery sample is more homogenous and ancestrally more similar to the target ALSPAC sample than the replication sample, which was a meta-analysis of multiple samples. Furthermore, the effects that are observed are very small (*R*^2^ ≤ .0034), albeit they are comparable to those reported in other studies using the composite genetic risk score method (Anney et al., 2012; Hamshere et al., 2013a). Small effect sizes in this type of study are affected by the relatively small ADHD GWAS discovery sample sizes, which have low power to detect susceptibility variants and thus a poor signal-to-noise ratio, making it unlikely that the current analysis reflects the true magnitude of the observed associations (Neale et al., 2010; Stergiakouli et al., 2012). However, the possibility of a false positive finding cannot be ruled out without additional replication.

As associations were observed only between ADHD composite genetic risk scores and IQ and working memory, but not inhibitory control and emotion recognition abilities, this suggests the possibility of specific pleiotropic effects. Interestingly, in children with ADHD, increasing levels of ASD traits are associated with lower IQ and more severe working memory difficulties, although not with attentional flexibility (Cooper, Martin, Langley, Hamshere, & Thapar, 2014). This previous study suggests that IQ and working memory problems may be a marker of additional neurodevelopmental problems or phenotypic complexity in the context of an ADHD diagnosis. The current study builds on this finding by suggesting that genetic risks for ADHD are relevant to lower IQ and working memory abilities in the general population, in addition to parent-reported neurodevelopmental behavioural traits (Martin et al., 2014b). The results are also comparable to a study of ADHD probands and their siblings, which found that the genetic factors contributing to low IQ are unlikely to explain the shared genetic effects of ADHD with other cognitive phenotypes such as performance on a go/no-go task (Wood et al., 2011).

Although the correlations between the neurocognitive abilities and ADHD and social-communication traits were modest, the results from this general population sample are in keeping with findings from clinical studies showing lower IQ, working memory, inhibitory control and emotion recognition abilities in children with ADHD and other neurodevelopmental problems, such as ASD (Frazier et al., 2004; Matson & Shoemaker, 2009; Uekermann et al., 2010; Uljarevic & Hamilton, 2012; Willcutt et al., 2008). Furthermore, the magnitude of these results is consistent with the known heterogeneity of neurocognitive abilities in children with ADHD; despite group differences, when compared with controls, not all children with ADHD or other neurodevelopmental problems experience these additional deficits (Nigg, Willcutt, Doyle, & Sonuga-Barke, 2005; Willcutt et al., 2008).

The results also show that on a population level, there are average differences in neurocognitive abilities between males and females, with males showing poorer working memory and emotion recognition abilities. It is well known that neurodevelopmental problems are also more common in males than females (Keen & Ward, 2004; Lichtenstein et al., 2010). However, the reasons for these gender group differences are unclear. One theory suggests that males have a lower liability threshold than females, which leads to them developing problems when exposed to a lower burden of risk variants. This theory has some support in terms of clinical ADHD and ASD phenotypes (Hamshere et al., 2013a; Rhee & Waldman, 2004; Robinson, Lichtenstein, Anckarsäter, Happé, & Ronald, 2013) but it is unknown whether it could also explain observed gender differences in neurocognitive domains. Further work is needed to explore the nature of these gender differences in neurocognitive abilities, particularly in the context of neurodevelopmental problems.

Given the longitudinal nature of the ALSPAC sample and the associated nonrandom attrition (Wolke et al., 2009), missing data were handled by using the ‘full information maximum likelihood’ (FIML) estimator in the SEM analyses. This approach maximised the use of all available data, in contrast to using pairwise deletion. A sensitivity test showed that restricting the SEM analyses to children who had complete data for each analysis, using listwise deletion, decreased the effect sizes, but otherwise did not alter the pattern of observed results (see Figure S7).

One additional limitation of this study is that the neurocognitive tasks assessed in the ALSPAC sample around ages 7–9 years (i.e. when the ADHD and social-communication measures were assessed), were not specifically selected for previously showing familial effects with ADHD. Previous family and twin studies have suggested that there are shared genetic effects between ADHD and measures of IQ, working memory (as assessed by the WISC Digit Span test), inhibitory control (assessed using a stop-signal reaction time task) and other cognitive measures not examined in the current study (Bidwell et al., 2007; Kuntsi et al., 2004; Schachar et al., 2005). At present it is not known whether some of the cognitive measures used in this study (i.e. the Opposite Worlds or DANVA tasks) share genetic risks with ADHD.

## Conclusion

Results from this population study indicate that a composite score of common genetic risk, previously found to be associated with a clinical ADHD diagnosis, predicts lower general cognitive ability and lower working memory in children in the general population. These genetic variants appear to have pleiotropic effects, predicting the presence of behavioural traits and neurocognitive performance in children. These results extend a growing body of literature highlighting the importance of shared molecular genetic factors across multiple psychiatric and psychological phenotypes.

**Key points**

- Attention-deficit hyperactivity disorder (ADHD) and other neurodevelopmental disorders (e.g. autism spectrum disorder (ASD) and schizophrenia) are highly heritable and may overlap in genetic risks.
- They are also associated with general cognitive delay and difficulties with executive functions and social cognition.
- A composite measure of common genetic risk variants relevant to a clinical diagnosis of ADHD has previously been found to predict traits of ADHD and social-communication difficulties in the general population.
- This study suggests that common genetic variants relevant to ADHD diagnosis may also play a role in normal variation in IQ and working memory abilities, independent of their effect on ADHD and ASD-like/social-communication traits in the general population.

## Supporting Information

**Figure S1.**Latent variable of neurodevelopmental outcomes (*N* = 6,434). **Figure S2.** Associations between composite genetic risk scores with inhibitory control and facial emotion recognition. **Figure S3.** Associations between composite genetic risk scores with IQ and working memory as correlated outcomes. **Figure S4.** Associations between composite genetic risk scores with ADHD traits and neurocognitive measures. **Figure S5.** Associations of neurocognitive phenotypes with ADHD composite score, using a variety of *p*-value selection thresholds. **Figure S6.** Association between composite genetic risk scores with working memory at age 10.5 years. **Figure S7.** Associations between composite genetic risk scores and neurocognitive measures, using listwise deletion. **Table S1.** Associations of composite genetic risk scores with neurocognitive outcomes, after adjusting for EIGENSTRAT covariates.
