# Supplementary material for: Neurocognitive abilities in the general population and composite genetic risk scores for attention-deficit hyperactivity disorder
Source: J Child Psychol Psychiatry. 2014 Oct 3;56(6):648–56. doi: 10.1111/jcpp.12336 (PMC4431584; doi:10.1111/jcpp.12336)
Supplement: Supplementary file 1 — Figure S1.Latent variable of neurodevelopmental outcomes (N = 6,434). Figure S2. Associations between composite genetic risk scores with inhibitory control and facial emotion recognition. Figure S3. Associations between composite genetic risk scores with IQ and working memory as correlated outcomes. Figure S4. Associations between composite genetic risk scores with ADHD traits and neurocognitive measures. Figure S5. Associations of neurocognitive phenotypes with ADHD composite score, using a variety of p-value selection thresholds. Figure S6. Association between composite genetic risk scores with working memory at age 10.5 years. Figure S7. Associations between composite genetic risk scores and neurocognitive measures, using listwise deletion. Table S1. Associations of composite genetic risk scores with neurocognitive outcomes, after adjusting for EIGENSTRAT covariates. [file jcpp0056-0648-sd1.docx]

**Supplementary Materials**

## **Figure S1 – Latent variable of neurodevelopmental outcomes (N=6,434)**


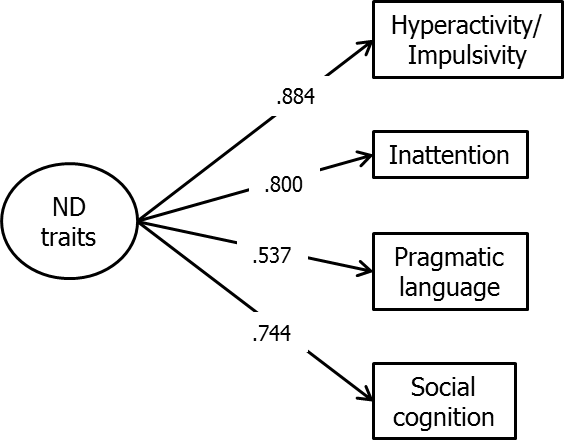


ND: Neurodevelopmental; Goodness of fit statistics: RMSEA=0.053, CFI=0.994, TLI=0.982

## **Figure S2 – Associations between composite genetic risk scores with inhibitory control and facial emotion recognition**

### Figure S2a


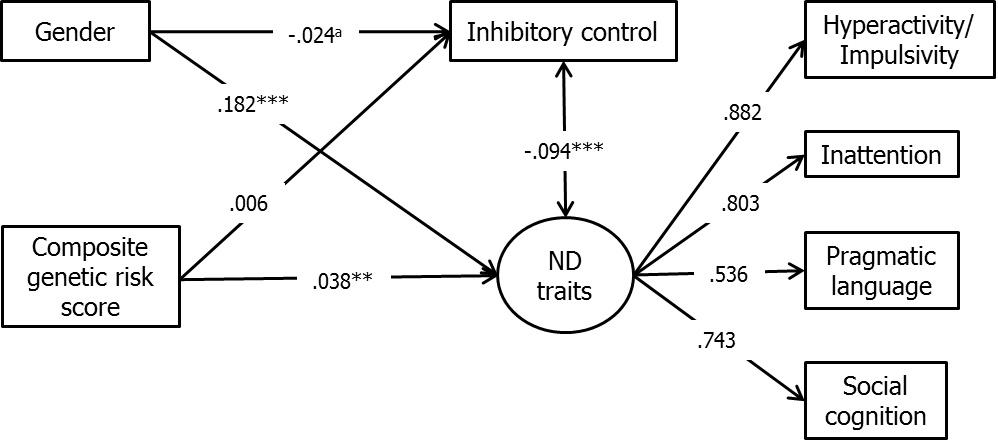


### Figure S2b


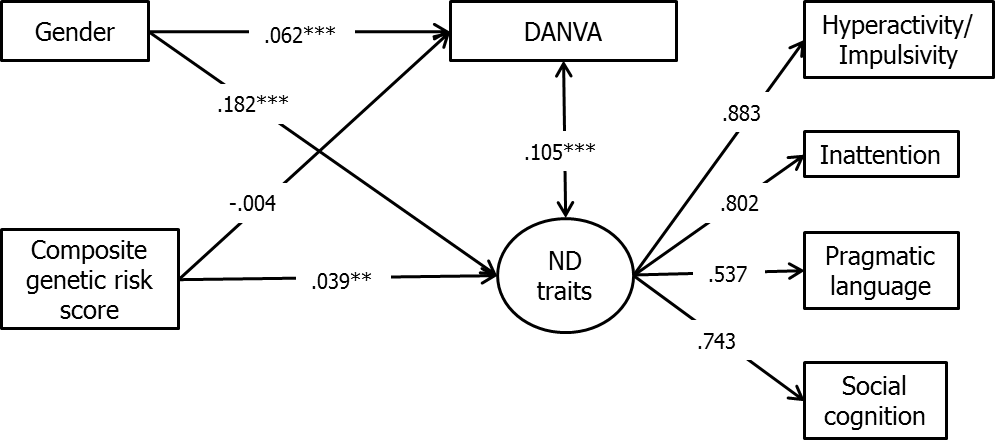


^a^ p<0.1, *p<0.05, **p<0.01, ***p<0.001; ND: Neurodevelopmental; DANVA: Diagnostic Analysis of Nonverbal Accuracy (facial emotion recognition task)

Fig. S2a: Association of composite genetic risk scores with inhibitory control (N=6,823); RMSEA=0.031, CFI=0.991, TLI=0.984. Fig. S2b: Association of composite genetic risk scores with facial emotion recognition (N=6,799); RMSEA=0.026, CFI=0.993, TLI=0.988

## **Figure S3 – Associations between composite genetic risk scores with IQ and working memory as correlated outcomes**


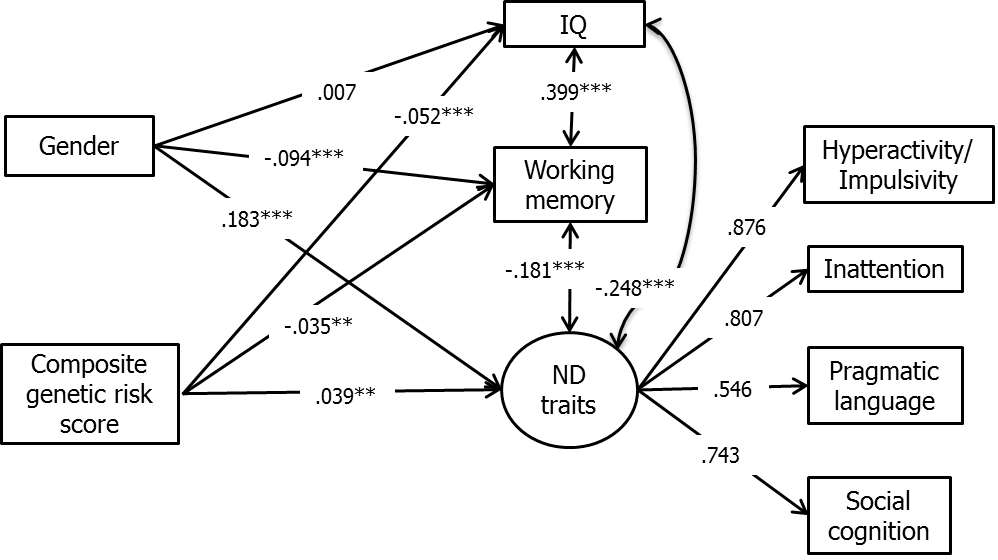


^a^ p<0.1, *p<0.05, **p<0.01, ***p<0.001; ND: Neurodevelopmental

N=6,835; RMSEA=0.052, CFI=0.973, TLI=0.948

## **Figure S4 – Associations between composite genetic risk scores with ADHD traits and neurocognitive measures as correlated outcomes**

### Figure S4a


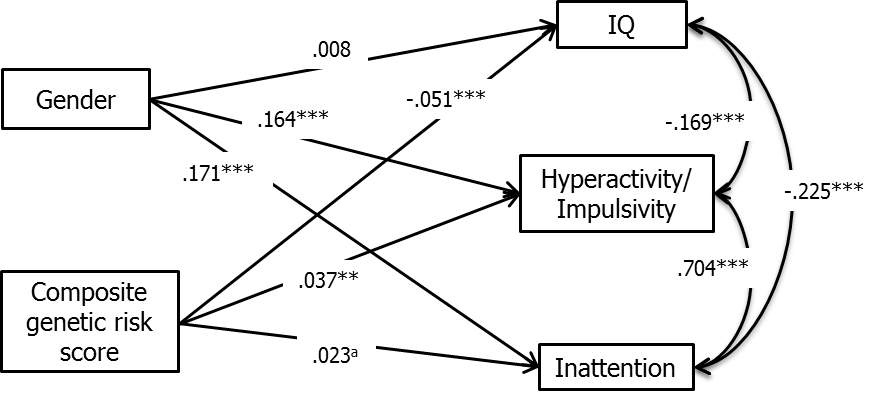


### Figure S4b


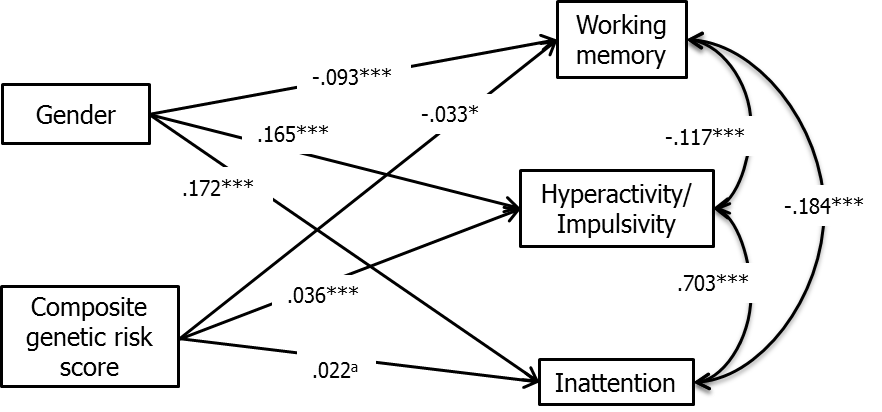


### Figure S4c


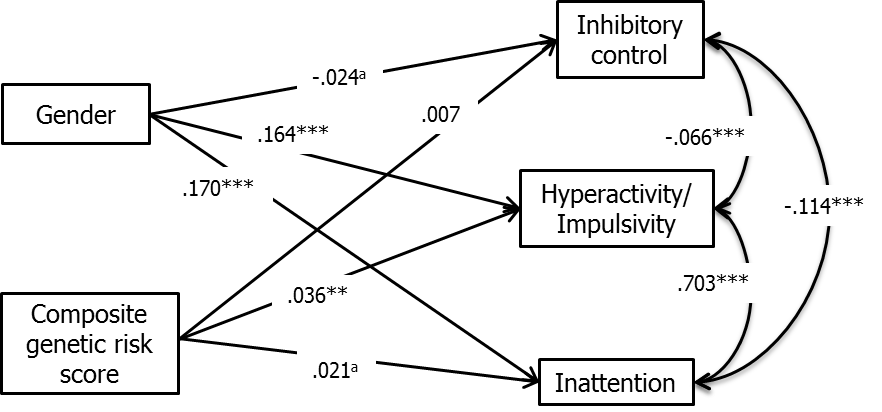


### Figure S4d


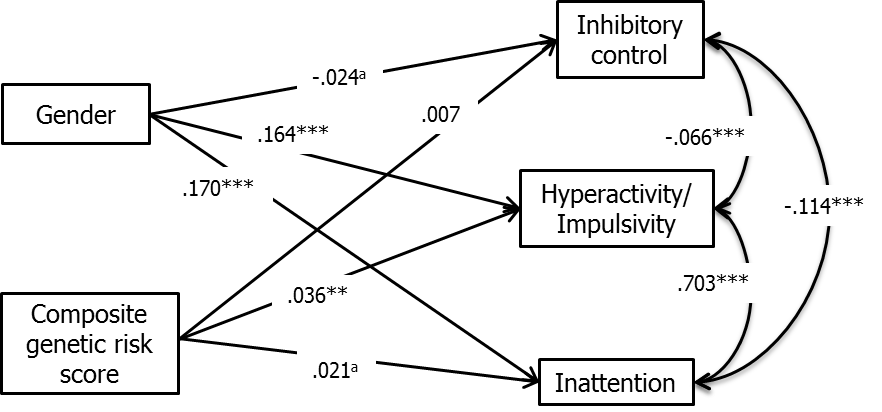


### Figure S4e


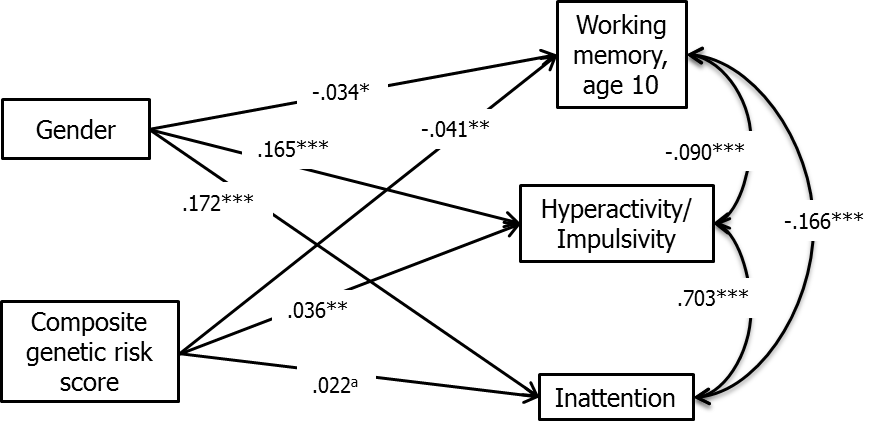


^a^ p<0.1, *p<0.05, **p<0.01, ***p<0.001; ND: Neurodevelopmental; DANVA: Diagnostic Analysis of Nonverbal Accuracy (facial emotion recognition task); No fit statistics available due to saturation of models

Fig. S4a: Association of composite genetic risk scores with ADHD traits and IQ as correlated outcomes (N=6,591). Fig. S4b: Association of composite genetic risk scores with ADHD traits and working memory, age 8.5 years (N=6,582). Fig. S4c: Association of composite genetic risk scores with ADHD traits and inhibitory control (N=6,575). Fig. S4d: Association of composite genetic risk scores with ADHD traits and facial emotion recognition (N=6,532). Fig. S4e: Association of composite genetic risk scores with ADHD traits and working memory, age 10.5 years (N=6,611).

## **Table S1 – Associations of composite genetic risk scores with neurocognitive outcomes, after adjusting for 10 EIGENSTRAT covariates and gender (linear regressions)**

| Outcome | N | β | p | R^2^ |
| --- | --- | --- | --- | --- |
| IQ | 5515 | -0.052 | <0.001 | 0.0027 |
| Working memory, age 8.5 years | 5411 | -0.033 | 0.014 | 0.0012 |
| Emotion recognition | 5107 | -0.004 | 0.80 | <0.0001 |
| Inhibitory control | 5315 | 0.005 | 0.72 | <0.0001 |
| Working memory, age 10.5 years | 5273 | -0.042 | 0.003 | 0.0017 |

## **Figure S5 – Associations of neurocognitive phenotypes with ADHD composite score calculated based on the primary discovery sample, using a variety of p-value thresholds (linear regressions)**


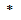
**
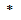

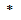

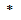

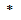

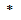

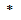

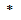

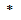
**
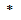

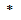

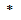

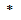

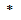

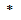

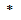

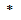

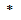

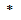

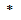

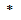

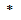

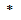

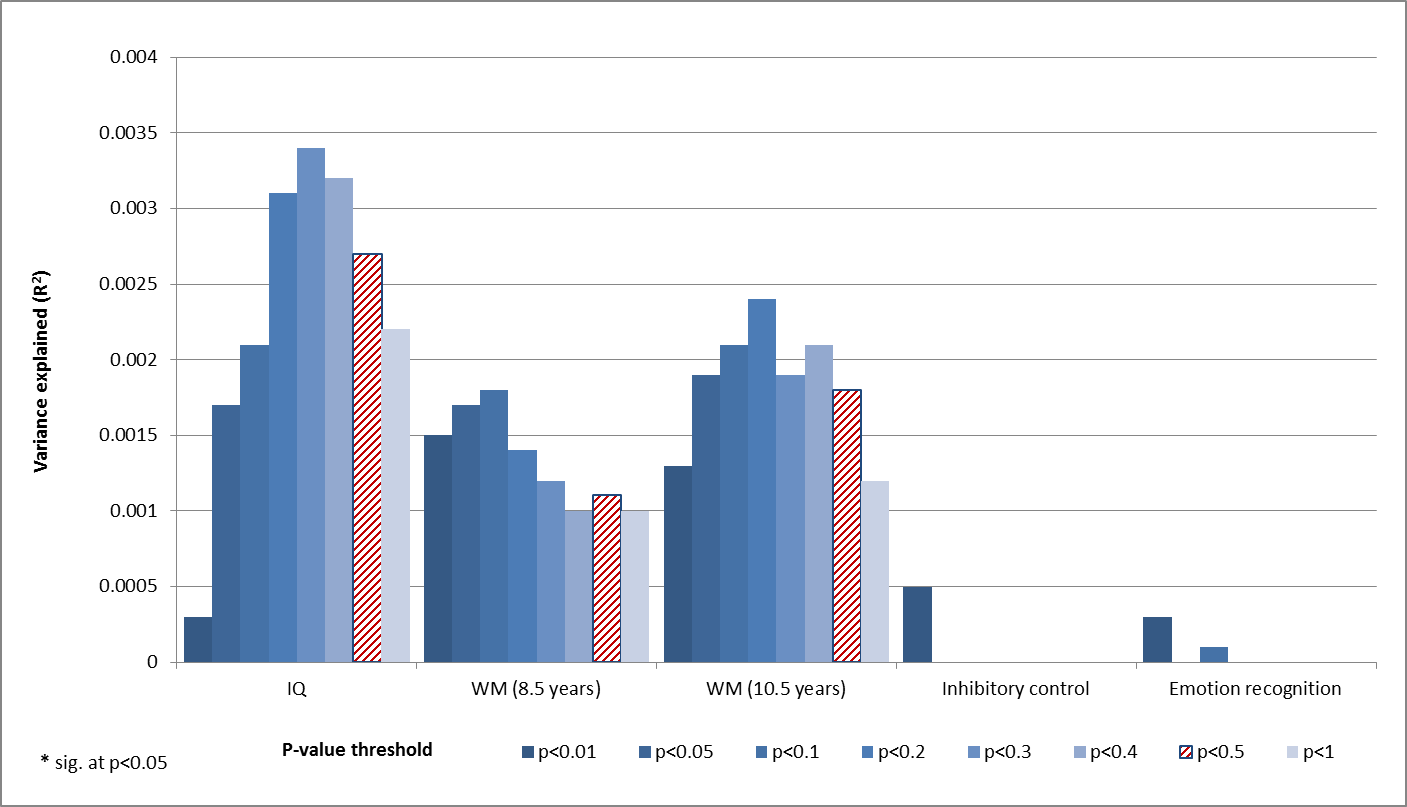


WM: working memory; Main results are based on composite genetic risk scores derived using a threshold of p<0.5 (striped bars).

## **Figure S6 – Association between composite genetic risk scores (based on the replication discovery sample) with working memory at age 10.5 years**


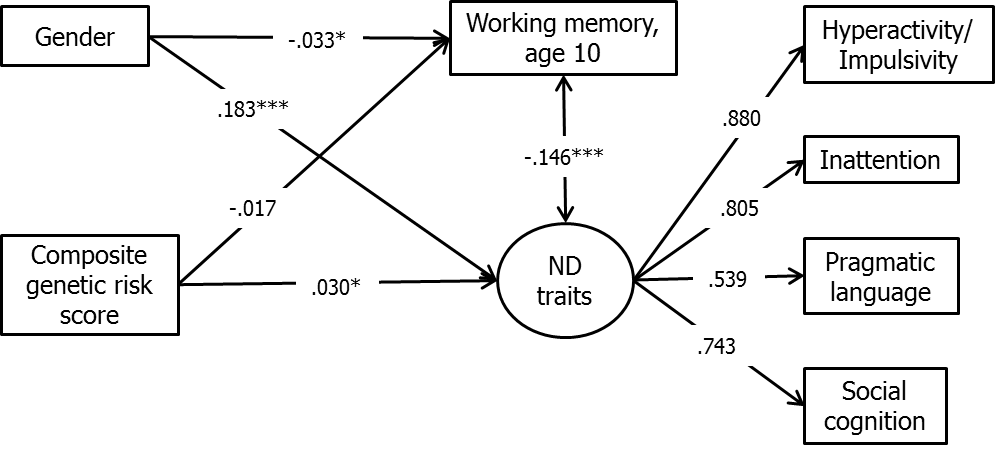


^a^ p<0.1, *p<0.05, **p<0.01, ***p<0.001; ND: Neurodevelopmental

Association of composite genetic risk scores, based on replication sample, with working memory at age 10.5 years (assessed with the Counting Span Task) (N=6,847); RMSEA=0.044, CFI=0.982, TLI=0.967

## **Figure S7 – Associations between composite genetic risk scores and neurocognitive measures, using listwise deletion**

### Figure S7a


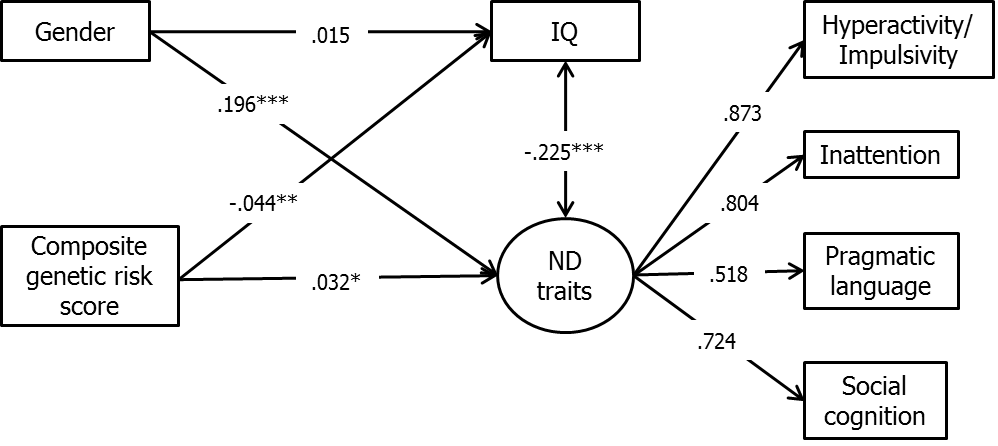


### Figure S7b


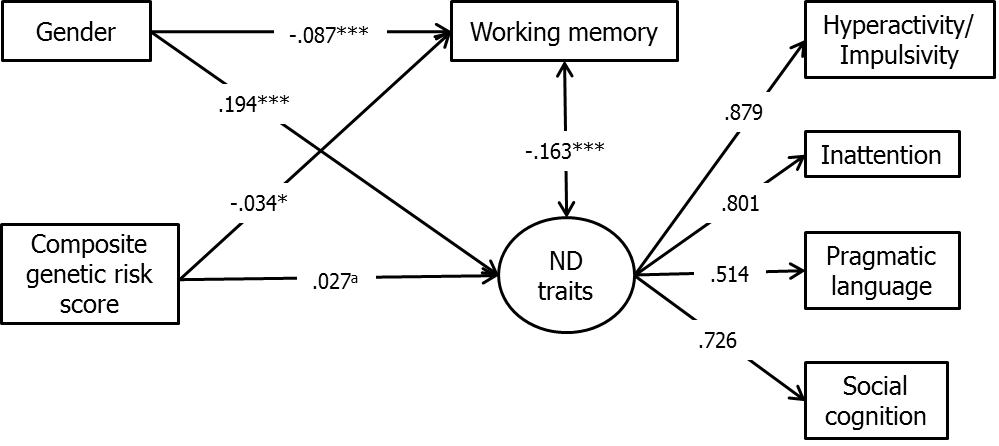


### Figure S7c


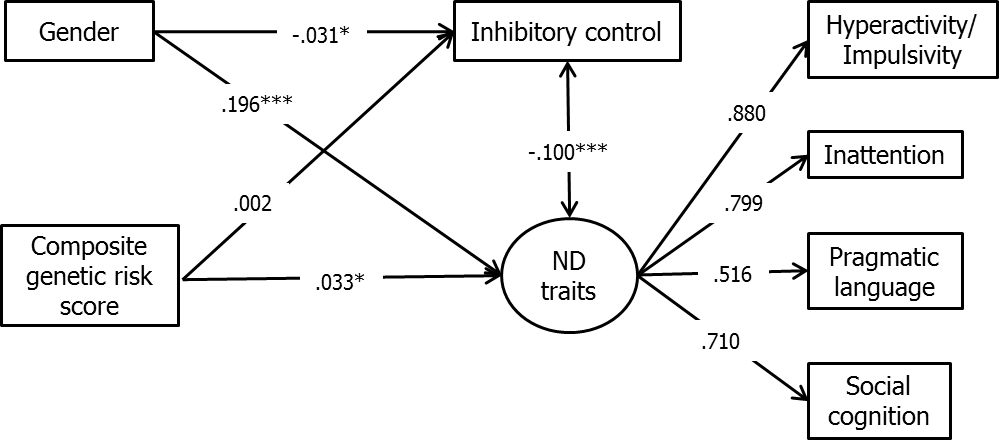


### Figure S7d


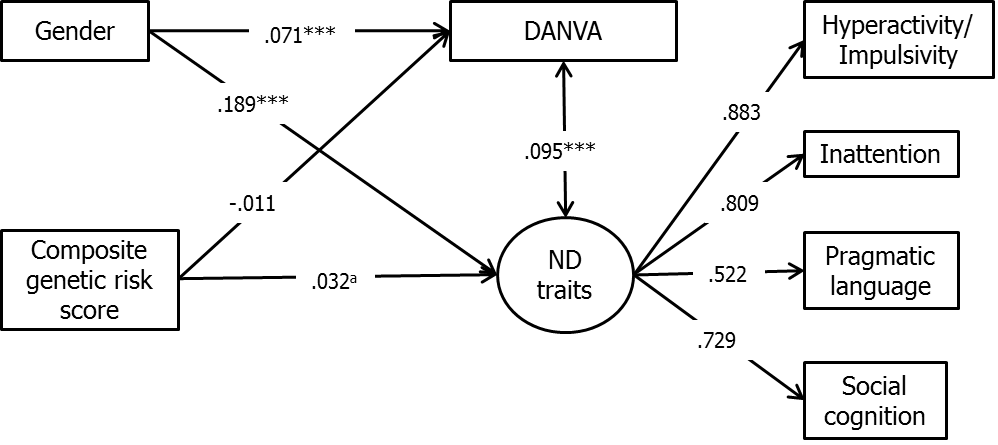


### Figure S7e


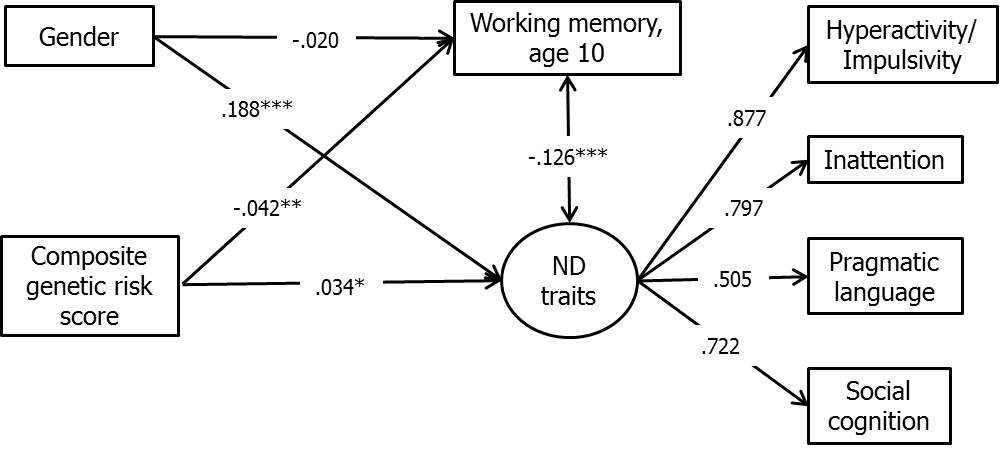


^a^ p<0.1, *p<0.05, **p<0.01, ***p<0.001; ND: Neurodevelopmental; DANVA: Diagnostic Analysis of Nonverbal Accuracy (facial emotion recognition task)

Fig. S7a: Association of composite genetic risk scores with ADHD traits and IQ as correlated outcomes (N=4,091); RMSEA=0.064, CFI=0.968, TLI=0.942. Fig. S7b: Association of composite genetic risk scores with ADHD traits and working memory, age 8.5 years (N=4,010); RMSEA=0.055, CFI=0.977, TLI=0.958. Fig. S7c: Association of composite genetic risk scores with ADHD traits and inhibitory control (N=3,928); RMSEA=0.039, CFI=0.988, TLI=0.978. Fig. S7d: Association of composite genetic risk scores with ADHD traits and facial emotion recognition (N=3,787); RMSEA=0.031, CFI=0.993, TLI=0.986. Fig. S7e: Association of composite genetic risk scores with ADHD traits and working memory, age 10.5 years (N=3,901); RMSEA=0.054, CFI=0.977, TLI=0.958.
